# Natural Products and Their Potential Anti-HAV Activity

**DOI:** 10.3390/pathogens10091095

**Published:** 2021-08-28

**Authors:** Agnieszka Jama-Kmiecik, Jolanta Sarowska, Dorota Wojnicz, Irena Choroszy-Król, Magdalena Frej-Mądrzak

**Affiliations:** 1 Department of Basic Sciences, Faculty of Health Sciences, Wroclaw Medical University, Chalubinskiego 4, 50-368 Wroclaw, Poland; agnieszka.jama-kmiecik@umed.wroc.pl (A.J.-K.); jolanta.sarowska@umed.wroc.pl (J.S.); irena.choroszy-krol@umed.wroc.pl (I.C.-K.); 2 Department of Medical Biology and Parasitology, Faculty of Medicine, Wroclaw Medical University, J. Mikulicza-Radeckiego 9, 50-345 Wroclaw, Poland; dorota.wojnicz@umed.wroc.pl

**Keywords:** HAV, essential oils, plant substances, foodborne viruses

## Abstract

The role of purified natural products in the prevention and treatment of countless diseases of bacterial, fungal, and viral origin cannot be overestimated. New antiviral drugs have been obtained from natural sources and transformed into preparations for prophylactic and therapeutic purposes. Flavonoids, polyphenols, saponins, proanthocyanins, polysaccharides, organic acids, proteins, polypeptides, and essential oils derived from plants, animals, or microorganisms can control and combat foodborne viral infections, including hepatitis A. The components of essential oils are characterized by numerous therapeutic and antioxidant properties and exhibit a broad spectrum of antimicrobial and antiviral activity. Due to these properties, they can be used to preserve meat, fruit, vegetables, and their products. Over the past two decades, much effort has been made to identify natural products, mostly of plant origin, to combat foodborne viruses. Natural plant extracts have several potential uses, not limited to increasing the safety of food products and improving their quality, but also as natural antiviral agents.

## 1. Introduction

Food contaminated with viruses can be a source of contamination for consumers, although foodborne viruses cannot multiply in food, unlike many microorganisms. Hepatitis A virus (HAV) has been associated with many recorded major outbreaks, while other human intestinal viruses such as human astrovirus (HAstV), human rotavirus (HRV), sapovirus (SaV), enterovirus (EV) and Aichi virus (AiV) have caused sporadic outbreaks around the world [1]. According to Yeargin et al., human intestinal viruses were detected in 13.1% and 45% of foodborne outbreaks in the EU and the USA, respectively [2].

The transmission of viruses to humans occurs through the consumption of contaminated food, through direct interpersonal contact, or through the environment, e.g., water [3]. Viruses can contaminate food at different stages of its production, from harvesting to post-harvest [4], and food ingredients can protect the virus during processing and human consumption. For example, fat, sugar, and protein in food can prevent viruses from deactivating [5,6]. The infectious dose of foodborne virus is generally low and a small quantity of virus particles can cause infection. Foodborne viruses can survive in food for long periods without losing infectivity [1]. Heat treatment is an effective means of deactivating foodborne viruses, but it can alter the organoleptic properties (e.g., color and texture) and reduce the nutrient content (e.g., protein and vitamins) of food [7]. Methods based on the use of heat as an effective method of pathogen deactivation are currently used in the food industry to combat bacteria and yeasts [8]. However, thermal deactivation of intestinal viruses, especially HAV in food, has not been fully understood [9,10,11,12]. Several studies have shown that heat treatment induces a conformational change in the capsid, leading to a reduction in initial viral titers, but the effect depends on the heat treatment conditions and the composition of the processed food [13,14]. A wide variety of food items, including drinks and juices, are usually preserved through heat treatment, which is characterized by a combination of two parameters: temperature and time. However, high-temperature treatment negatively affects food quality, ultimately reducing both the nutritional and sensory value of food [15,16].

Foodborne virus outbreaks are frequently associated with minimally processed and ready-to-eat foods, which is why alternative preservation methods are needed to inactivate viruses. It should also be noted that companies responsible for processing food should consider whether innovative non-thermal food processing technologies to deactivate viruses can also deactivate bacterial pathogens, such as Listeria monocytogenes, which may potentially survive in food during cold storage [17].

Gut viruses have received less attention than other foodborne pathogens, and strategies to combat them are necessary to ensure food safety and reduce the number of infections in humans.

Today, vegetables and berries are most commonly associated with intestinal virus outbreaks as they are generally eaten fresh or mildly processed (e.g., frozen or freeze-dried blueberries, blanched vegetables), increasing the risk of infection. These foods are prone to contamination due to the use of fecally contaminated water for irrigation or the lack of proper hygiene among food contact persons [18,19].

The consumption of fresh vegetables has increased significantly around the world with the advent of new technologies, and a wide range of products, from packaged salads to freshly cut fruit, makes the consumption of fresh fruit and vegetables affordable and convenient for consumers.

The consumption of fresh produce is part of a healthy diet, but pathogenic contamination has serious public health implications. Outbreaks of infections are often associated with tomatoes, spinach, lettuce, and seed sprouts. Vegetables can become contaminated with human pathogens at several points along the production chain [20].

Clear strategies for identifying sources of pathogens should be developed and steps taken to prevent contamination of vegetables along the entire production chain [21]. Sources of plant contamination have been broken down into pre-harvest (focused on improving soil quality, irrigation water, climate change, and geographic location) and post-harvest (harvesting, handling, and processing).

Potential sources of pathogen contamination prior to harvest include soil, wildlife feces, soil alteration, agricultural water, reconstituted fungicides and insecticides, dust, wild or domestic animals, field workers, and harvesting equipment [20,22,23]. Research shows that animal manure used to improve soil can contain viruses, parasites, or bacteria, which pose a risk to human health.

Irrigation is considered to be one of the most important ways of transmitting intestinal human intestinal pathogens to vegetables [24].

It is known that plants produce secondary metabolites with antimicrobial activity in response to pathogen attack as a defense mechanism. Today, there has been a significant increase in the interest of usage of natural products as effective antiviral therapeutic agents. This paper discusses the action of selected essential oils and natural products against hepatitis A virus. Recent in vitro study revealed that green tea extract (GTE) demonstrated excellent antiviral activity against hepatitis A virus under controlled conditions of concentration, pH, temperature, and also time exposure. It now appears that grape seed extract (GSE) exhibits rather greater antiviral activity by potentially blocking host cell receptors and consequently preventing virus entry, replication, and infection, while not causing any structural damage to the HAV viral capsid. In turn, proanthocyanidins from blueberries slightly reduce HAV replication in the host cells, but they interrupt HAV binding and penetration to a much greater extent. Research into natural anti-HAV products is promising as several have shown remarkable potential for controlling HAV infection and replication. These natural agents have been shown to exhibit significant antiviral activity against HAV and can act at different stages of the virus life cycle, replication, assembly, release, as well as targeting specific virus–host interactions.

The aim of the study was to evaluate, based on the available literature, the effect of essential oils and plant substances on the growth and survival of HAV.

## 2. Hepatitis A Virus

Hepatitis A virus (HAV), the cause of hepatitis A, is responsible for approximately 1.5 million reported cases and tens of millions of infections each year. Although hepatitis A is a self-limiting disease of the liver, it sometimes progresses to a severe, life-threatening form. HAV is a non-enveloped, single-stranded, positive polarity RNA virus belonging to Enteroviruses, genus Hepatovirus, family Picornaviridae, and can survive for several weeks in water or sewage. The HAV virus is resistant to external factors (temperature, chemicals, e.g., acids). It is stable in an environment with a high degree of acidification (pH 3) for about 4 h, which makes it resistant to digestive enzymes, which helps to survive the passage through the stomach on the way to another replication site in the small intestine. It is deactivated by autoclaving, boiling, UV radiation, disinfectants containing chlorine, or formalin. The viral particle is spherical, non-enveloped, 27 nm in diameter [25,26].

### 2.1. Genotypes of HAV

The HAV genome is approximately 7500 nucleotides, consisting of untranslated regions and a coding region. The coding region encodes a large polyprotein and has been divided into P1, P2 and P3 segments. P1 is translated into the three major viral capsid proteins, VP1, VP2, VP3 and VP4, which plays a role in virion formation. Non-structural proteins are generated by cleavage of P2 and P3 by proteases. The genome is heterogeneous, which allows the classification of HAV into six genotypes and several sub-genotypes. Heterogeneity has a limited effect on antigen diversity and results in the existence of single serotypes. So far, six genotypes have been identified (I, II, III, IV, V and VI) [27]. The most frequently isolated genotypes in humans are I and III. On the other hand, three consecutive genotypes (IV, V and VI) occur in monkeys of the macaque family (Cercopithecidae). Within genotypes I, II and III, sub-genotypes A and B are distinguished. HAV genotypes and sub-types do not occur evenly all around the world, and genotype I is the most common. In areas characterized by low endemicity, such as North America and Europe, the most common is sub-genotype IA. Furthermore, other genotypes and sub-genotypes, such as IB and IIIA, are also isolated in Europe. The IA and IB subtypes are widespread in North and South America, Europe, China, Japan, and Thailand. Most of the human strains beyond genotype I belong to sub-genotype IIIA and are found in India, Kazakhstan, Europe, and the USA. The most common sub genotype in the USA is IA, followed by the IB and IIIA sub-genotypes [28].

Recent studies have shown that HAV is evolutionarily different from other picornaviruses, not only in the nucleotide sequence of its genome, but also in the structure of its capsid, which shares features with primitive insect viruses. Although for many years only humans and other primate species were susceptible to infection with hepatoviruses, numerous distinct species of hepatovirus closely related to human HAV have recently been identified in various species of small mammals and are now classified under the same genus. Unlike many other picornaviruses, including the well-studied poliovirus, HAV does not block cellular protein synthesis in infected cells and generally replicates without a cytopathic effect. One of the most interesting functions of the virus is its recently recognized non-lytic ability to be released from infected cells in a membrane-wrapped form as infectious quasi-enveloped virions (eHAVs). eHAV particles are similar in infectivity to the non-enveloped virion and represent the second form of infectious virus [29].

### 2.2. Food Sources of HAV

Hepatitis A is mainly infected by the fecal–oral route (“dirty hands” disease). Infection can occur through direct contact with an infected person, consumption of contaminated food, consumption of contaminated water (also in the form of ice cubes), washing hands in contaminated water or following sexual contact with an infected person. Foods involved in the transfer of HAV include vegetables, fruits (fresh or frozen, especially berries), reconstituted frozen orange juice, shellfish, salads, sandwiches, ice cream, cheese, rice pudding, frozen dough, pudding, milk, bread, biscuits, and other raw or undercooked foods. Food may become contaminated with HAV in several different ways: fruits and vegetables grown and/or irrigated with fecally contaminated materials, crustaceans grown in and harvested from dung waters, food processing and preparation using fecally contaminated equipment or machinery, and handling of ready-to-eat food products by infected persons with poor personal hygiene. Food establishments with poor sanitation and inadequate waste disposal systems, along with unsatisfactory manufacturing practices, can also contribute to food contamination [30,31]. Frozen fruit has been shown to be a major carrier of foodborne illness attributed to hepatitis A virus (HAV) infections. Fresh products can become infected with viruses through direct contact with a contaminated surface, water, or hands, and then frozen without proper sanitation. Due to their structural integrity, foodborne viruses are able to withstand hostile conditions such as drying out and freezing and survive for long periods without losing infectivity. In addition, these foods are often eaten raw or undercooked, increasing the risk of infection. The Nasheri results revealed that frozen fruit, especially blueberries and pomegranate shells, contributed to most of the outbreaks, with outbreaks frequently reported in industrialized countries [32]. Analyses of the persistence of HAV RNA in water, on non-porous surfaces, and on blueberries were performed. A study by Trudel-Ferland showed that RNA associated with deactivated HAV persists significantly over time on non-porous surfaces and on foods over a wide temperature range. Stability at above freezing temperatures is also a cause for concern, although less so [33].

### 2.3. Epidemiology of HAV

The time from infection to the onset of symptoms may be 15 to 50 days (average 30 days). An infected person sheds large amounts of the virus in their feces for up to several weeks, and shedding may begin 2 to 3 weeks before the onset of jaundice, that is, while the person appears to be healthy. The excretion of the virus in the feces may take up to 6 weeks following infection. Prolonged viral excretion via the feces occurs mainly in children, while in immunocompetent adults the amount of virus excreted in the feces drops sharply after the onset of jaundice. The virus is also found in the blood and saliva of an infected person. Household members and sexual partners of infected people, intravenous drug users, and those traveling to endemic areas of hepatitis A are at increased risk of infection [34].

HAV infection is asymptomatic or with mild symptoms or symptoms varying in intensity from flu-like symptoms (in the first stage) and gastrointestinal symptoms—belching, heartburn, nausea, flatulence, early satiety, dark urine—to jaundice (i.e., yellowing skin, whites of the eyes, or mucous membranes). Acute inflammation and damage to the liver parenchyma may occur. In adults, the disease has a sudden onset and is severe, requiring hospitalization, occurring in the form of jaundice in 70% of people. In children up to 5 years of age, illnesses are rare, and if they do occur, the disease is asymptomatic or very mild. Over 70% of children under 6 years of age and 20% of children over 6 years of age have asymptomatic HAV infections. The severity of the disease is higher in the older age groups [34]. HAV complications are dangerous and can even lead to death. Complications of hepatitis A include hyperacute hepatitis and bone marrow aplasia. Hepatitis A virus does not cause chronic hepatitis, and immunity after falling ill is life-long. The long incubation period of viruses such as HAV makes it difficult to accurately determine the food product that is the source of infection for the people infected.

In animal models, HAV antigens can be detected in the stomach, small intestine, and large intestine throughout the duration of the infection, suggesting that some degree of viral replication is present in the gut. HAV reaches the liver via systemic circulation and infects hepatocytes. On entry into a cell, HAV interacts with HAVCR1, with a receptor attachment present on the cell surface. Additionally, HAVCR2 and the asialoglycoprotein receptor have been reported to participate in HAV entry into the cell. After replication in the liver, the virus is released into the bile and eventually excreted in the feces [35].

It is believed that the course of viral hepatitis is genetically determined by the variability of the hosts, whilst infection with the same virus leads to different disease courses of individuals. The molecular mechanisms responsible for the wide range of disease severity caused by HAV infection are not well understood. It is widely accepted that virulence factors associated with specific viral lines and various host factors such as age, sex, and race play a role in the clinical outcomes of HAV infections [35].

**Figure 1:**
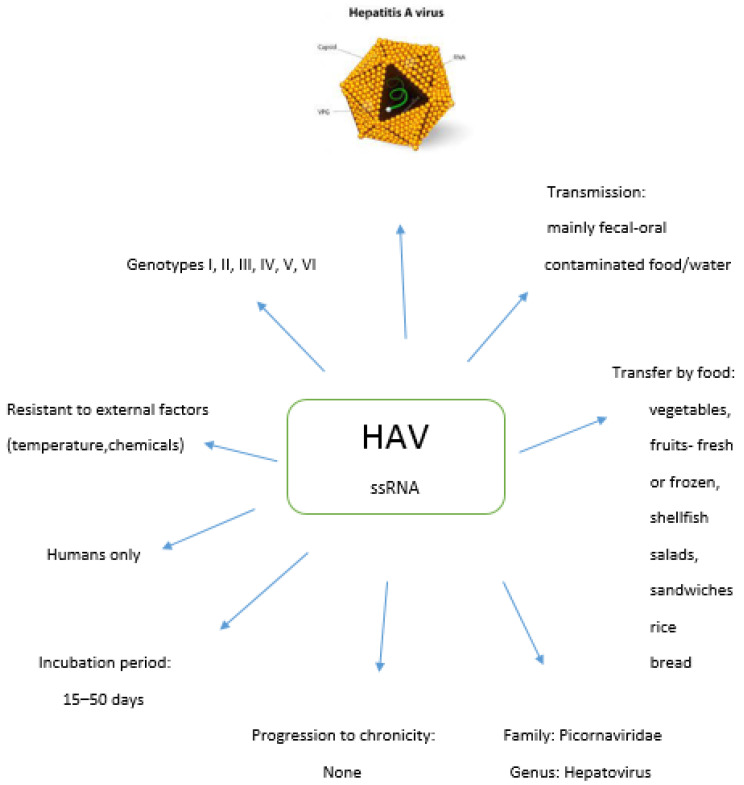
Characteristics of HAV.

It is estimated that almost 80% of the population aged 30–55 is non-immune. The elderly over 70 years of age are immunized after exposure to the virus, but only 6.5% of adults up to 40 years of age have HAV antibodies. Figure 1 shows the characteristics of HAV.

Annually, between 1.2 and 1.4 million cases of the disease are recorded, although there are actually many more cases, as it is estimated that only every tenth case of the disease is reported. Overall, the WHO estimated that 7134 people worldwide died from hepatitis A in 2016 (corresponding to 0.5% of the death rate from hepatitis). Among the sick, 20% require hospitalization, and mortality is low, at 0.6–2.1%. HAV infections most often occur in areas of poor sanitary conditions, among people who do not observe good personal hygiene. The countries with high endemic incidence of HAV include Bulgaria, Egypt, Tunisia, countries of the Mediterranean basin and Africa, countries of Eastern Europe, and Russia [25,26,36].

Foodborne HAV outbreaks have previously occurred concerning, for example, infections caused by the consumption of frozen or fresh blueberries and sun-dried tomatoes, as well as oysters and other crustaceans. Food contact can be another source of HAV, as HAV is environmentally stable and can remain contagious for long periods in the environment and on inanimate surfaces. An epidemic, recorded in Germany in 2017–2018, was caused by one of the HAV strains identified from those infected with an MSM group combined with a contact person working in a food store as the most likely source of the outbreak. Through contact tracing, two direct contacts were diagnosed by stool examinations, and over the next few weeks, seven of the store’s customers were identified as confirmed cases [34].

Based on data published by ECDC in 2018, a total of 42 atypical cases of hepatitis A were reported in six European Union (EU) countries. They were infected with one of two different strains of hepatitis A virus (HAV) genotype IA. The cases were classified as either indigenous, i.e., infected in the EU, or travel-related, i.e., travel history to Morocco. Both HAV strains have historically been found to be epidemiologically linked to Morocco. However, many of the 2018 cases have no history of travel to Morocco. These cases were identified by sequencing the viral RNA fragment in the VP1/P2A overlap region. HAV strains with one to two nucleotide differences in this RNA region are likely to have a common ancestry. Based on the molecular findings on returning Moroccan travelers and residents of Morocco, it is likely that these strains have been circulating in Morocco since at least 2011 and this transmission in Morocco continued until recently.

In EU countries in 2012–2014, strains associated with two foodborne disease outbreaks were related to the consumption of frozen strawberries and frozen mixed berries [26,36,37,38]. In 2016–2018 in the USA, the number of hepatitis A infections increased by 294% compared to 2013–2015; they were mainly associated with outbreaks related to contaminated foodstuffs, among MSM, and mainly among those reporting drug use or homelessness [39].

## 3. Composition and Biological Activity of Essential Oils

Essential oils (EOs) are liquid, volatile fragrances which are most often obtained by steam distillation from a suitable plant material. They are a mixture of various compounds, such as ketones, aldehydes, alcohols, esters, lactones, terpenes, and organic compounds. The components of essential oils are characterized by numerous therapeutic and antioxidant properties and exhibit a broad spectrum of antimicrobial and antiviral qualities. Due to these properties, they can be used to preserve meat, fruit, vegetables, and their preserves.

The available literature provides information on the use of various EOs against hepatitis A virus (HAV). These oils include oregano oil, thyme oil, *Zataria multiflora* oil, lemon oil, sweet orange oil, grapefruit oil, rosemary oil, *C. indicum* oil, and *C. morifolium* oil.

The main components of oregano EO extracted from *Origanum vulgare* are carvacrol (0.3–80.8%), belonging to the monoterpenes; and thymol (0.96–63.7%), belonging to the sesquiterpenes. The other ingredients present in lower proportions are gamma-terpinene (0.8–21.0%), P-cymene (<0.1–16.94%), alpha-terpineol (<0.09–12.0%), and limonene (0.3–0.7%) [40,41]. Numerous in vitro and in vivo studies have shown the antibacterial, antiviral, and antifungal effects of the oregano EO. This essential oil was shown to possess antibacterial activity against Gram-negative bacteria: *Escherichia coli*, *Salmonella choleraesuis*, *Salmonella Typhimurium*, *Shigella sonnei*, *Pseudomonas aeruginosa*, *Klebsiella pneumoniae*, *Klebsiella oxytoca* and Gram-positive bacteria: *Clostridium botulinum*, *Clostridium perfringens*, *Listeria monocytogenes*, *Staphylococcus aureus*, *Bacillus subtilis*, *Sarcina lutea*, and *Mariniluteicoccus flavus* [42]. The oregano essential oil was also shown to be effective against fungal pathogens—*Candida albicans*, *Malassezia furfur*, *Trichophyton rubrum*, *Trichosporon beigelii*, *Aspergillus niger*, and *Aspergillus tubingensis* [43,44,45,46]. Due to antimicrobial, antifungal, and antioxidative properties oregano essential oil can be used as a food preservative [47]. Studies have shown that these properties are related to the presence of carvacrol and thymol [48].

The main ingredient of the essential oil obtained from *Thymus vulgaris* is thymol belonging to sesquiterpenes (27.6–100%). The other ingredients of thyme oil are trans-sabinene hydrate (0.43–39.4%), menthol (1.3–39%), bornyl acetate (0.2–25.57%), limonene (0.4–24.2%), carvacrol (2.0–20.5%), and gamma-terpinene (0.6–14.9%) belonging to monoterpenes [40,49,50]. The research results presented by Borugă et al. 2014 showed the effectiveness of thyme EO against the food-related bacteria and fungus—*S. aureus*, *P. aeruginosa*, *S. Typhimurium*, *E. coli*, *K. pneumoniae*, *E. faecalis*, and *C. albicans*. This antimicrobial property of EO could be attributed to the presence of the major constituent—thymol. The antioxidant properties of the *T. vulgaris* EO allow it to be used as a food additive as a means of protecting it from spoilage [51].

The main active substances in the essential oil obtained from *Zataria multiflora* Boiss are thymol (40.8%), belonging to sesquiterpenes; and carvacrol (27.8%), belonging to monoterpenes. P-cymene—alkylbenzene related to a monoterpene (8.4%), sesquiterpene—beta-caryophyllene (2.0%), and monoterpenes: gamma-terpinene (4.0%), linalol (1.7%), and alpha-terpinolene (1.3%) are the remaining ingredients of the oil [40,52]. It was found that *Z. multiflora* EO has antimicrobial properties. The growth of *P. aeruginosa*, *K. pneumoniae*, methicillin-resistant and methicillin-sensitive *S. aureus* strains was inhibited in the presence of EO [52,53,54,55]. The key ingredients, carvacrol and thymol, are responsible for the antimicrobial and antioxidant properties of *Z. multiflora* EO [55]. This EO exhibits positive antioxidant, antibacterial, and antifungal properties, and can therefore function as an effective preservative in food—for example, in cookies or hamburgers [56,57].

Limonene, beta-pinene, and gamma-terpinene, belonging to monoterpenes, are the key components of the essential oil derived from lemon essential oil (*Citrus limon*). Their content is 61.09–71.18%, 10.03–13.41%, and 7.73–9.89%, respectively. In much smaller quantities are geranial (1.9–2.4%) and neral (1.39–1.77%), belonging to monoterpene aldehydes [40,58,59]. Lemon EO showed antibacterial properties against Gram-positive strains: MRSA, MSSA, *E. faecalis*, *L. monocytogenes*, *B. subtilis* and Gram-negative strains: *E. coli*, *K. pneumoniae*, and *P. aeruginosa* [58,60,61]. The antifungal activity of lemon oil against some food spoilage fungal species, especially *Aspergillus* and *Penicillium species*, was also described [62]. *C. limon* EO can be use in the prevention of contamination and growth of pathogenic bacteria during minced beef meat storage at 4 °C [58]. Ajayi-Moses et al. 2019 [63] noted that lemon EO exhibited pronounced inhibitory potential against microorganisms associated with fruits spoilage; therefore, it can be used as a preservative agent.

The main ingredients of sweet orange EO derived from *Citrus sinensis* are limonene (92.1–95.9%) and linalool (0–5.6%) [40,59,64]. Sweet orange EO was reported to inhibit the growth of Gram-positive and Gram-negative bacteria, including *S. aureus*, *L. monocytogenes*, *Vibrio parahaemolyticus*, *S. Typhimurium*, *E. coli*, and *P. aeruginosa* [65,66,67], as well as several fungal species, such as *Aspergillus flavus*, *Aspergillus fumigatus*, *Aspergillus terreus*, *A. niger*, *Alternaria alternata*, *Cladosporium herbarum*, *Curvularia lunata*, *Fusarium oxysporum*, *Helminthosporium oryzae*, *Penicillium chrysogenum*, *Penicillium verrucosum*, and *Trichoderma viride* [68,69,70]. Sweet orange EO exhibits antioxidant, antifungal, and antibacterial properties, which have important applications in food industries [71,72].

Limonene is the main ingredient in grapefruit EO (*Citrus paradisi*). Its content ranges from 84.8 to 93.45%. The remaining ingredients are present in small quantities: myrcene (6.9%) and alpha-pinene (1.7%), belonging to monoterpenes; and beta-caryophyllene (1.1%), belonging to sesquiterpenes [40,59,73]. *C. paradisi* EO inhibited the growth of *E. coli*, *S. aureus*, *E. faecalis*, *S. Typhimurium*, *Lactococcus lactis*, *Leuconostoc mesenteroides*, *Lactobacillus plantarum*, *Staphylococcus epidermidis*, *Serratia marcescens*, and *Proteus vulgaris* [73,74,75]. The effectiveness of *C. paradisi* EO against fungi: *A. niger*, *A. flavus*, *C. albicans*, *Penicillium chrysogenum*, *Fusarium moniliforme* and *Saccharomyces cerevisiae* has been confirmed [76]. According to Luciardi et al. 2019 [77], grapefruit essential oils could be used as a food preservative to control *P. aeruginosa* virulence.

The major constituents of essential oils from *Rosmarinus officinalis* are 1,8-cineole (16.0–58.6%), alpha-pinene (2.5–48.0%), and camphor (1.4–26.0%). Much smaller amounts of alpha-terpineol (0.7–12.8%), beta-caryophyllene (0.5–13.6%), borneol (1.0–9.0%), camphene (1.7–7.0%), limonene (1.8–5.4%), and beta-pinene (0.7–3.8%) are also present in the oil [40,59,78]. EO from *R. officinalis* has been demonstrated as having antibacterial properties against *E. coli*, *P. aeruginosa*, *S. aureus*, *B. subtilis*, *Bacillus cereus*, *Bacillus pumilis*, *Clostridium perfringens*, *Aeromonas hydrophila*, *Salmonella choleraesuis*, and *Salmonella poona* [79,80]. Rosemary EO exhibited fungitoxic activity against *Penicillium* spp., *A. niger*, and *A. flavus* [81,82,83]. Due to its antimicrobial and antioxidative properties, rosemary EO can be successfully used as a food preservative, especially in foods that contain animal or vegetable fats [84].

*Chrysanthemum indicum* essential oil contains camphor (7.75–36.69%), bornyl acetate (10.00–21.33%), and borneol (3.3–18.34%) as the major constituents followed by 1,8-cineole (0.12–10.4%), alpha-terpinene (5.73%), and caryophyllene oxide (0.13–5.46%) [85,86,87]. The essential oil of *C. indicum* exhibited stronger antibacterial activity against all oral bacteria: *Streptococcus mutans*, *Streptococcus sanguinis*, *Streptococcus sobrinus*, *Streptococcus ratti*, *Fusobacterium nucleatum*, *Prevotella intermedia*, and *Porphylomonas gingivalis*. *E. coli* and *S. aureus* strains appeared to be less sensitive to *C. indicum* EO [85]. Shunying et al. 2005 demonstrated the strong activity of the essential oil against clinical (*E. coli*, *K. pneumoniae*, *Staphylococcus saprophyticus*, *Enterobacter cloacae*) and reference (*B. subtilis*, *S. aureus*, *Salmonella typhi*) bacterial strains. Fungi: *Candida* spp. and *Hansenula anomala* were also sensitive to *C. indicum* EO [86]. The anti-infectious and antioxidant properties of *C. indicum* EO make it suitable for use as a food spice to preserve it and protect it from deterioration [87].

**Figure 2:**
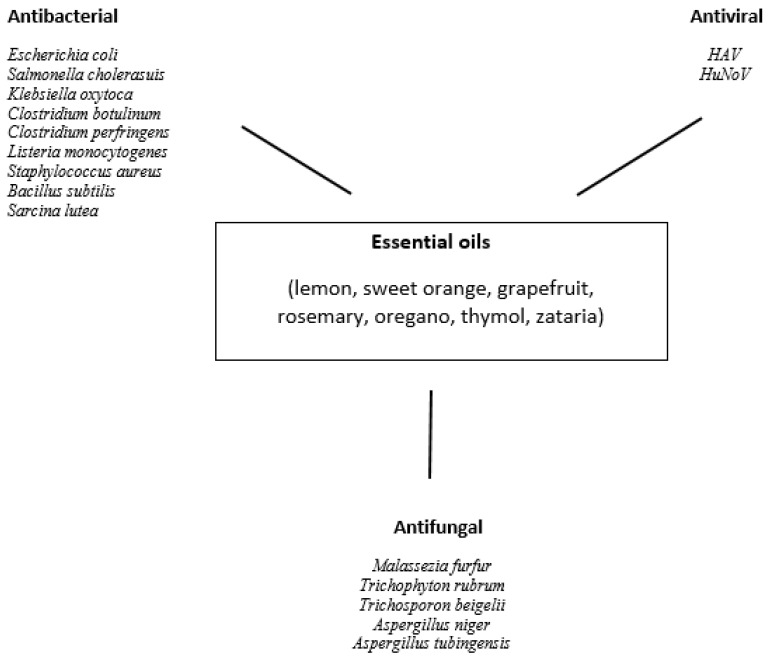
Effects of essential oils on microorganisms.

Chrysanthenone was also the predominant component of *Chrysanthemum morifolium* essential oil (9.71–48.96%). The other ingredients of the oil are verbenone (17.33%), camphor (14.56–44.6%), curcumene (10.50%), eudesmol (8.92%), pentacosane (8.65%), borneol (7.95%), and copaene (5.61%) [87,88,89]. The essential oil of *C. morifolium* exhibits significant inhibitory effects toward *E. coli, S. aureus, S. epidermidis, S. Typhimurium, S. sonnei, Shigella flexneri, Citrobacter freundii, Streptococcus agalactiae, Streptococcus pyogenes*, and *Pseudomonas fluorescens* [87,89]. Bacteria *P. aeruginosa* and *K. pneumoniae* were resistant to the essential oil [89]. Other research suggests that secondary metabolites of *C. morifolium* possess antifungal activity against *F. oxysporum, Magnaporthe oryzae*, and *Verticillium dahlia* [90]. *C. morifolium* EO, just like *C. indicum* EO, can be used as a food preservative due to its antimicrobial and antioxidative properties [87]. The effect of essential oils on microorganisms is shown in Figure 2.

## 4. Review of Antiviral Use of Essential Oils, Juices, and Other Plant Extracts against HAV

Essential oils are volatile substances which are naturally produced by plants, and are obtained from parts of plants such as flowers, buds, stems, leaves, seeds, twigs, roots, fruits, bark, and wood. They are produced by plants as secondary metabolites and are valuable for their antibacterial, antifungal, insecticidal, and antiviral properties [91]. Essential oils are used in the production of cosmetics and in the perfumery and pharmaceutical industries.

In the studies of Battistini et al. [59], the HAV virus was multiplied in Frp3 cells. Virus titer was determined using the Reed and Muench method to set the 50% dose of infectious tissue culture (TCID50). Four oils in various concentrations were used in the research: lemon EO (0.5%), sweet orange EO (0.1%), grapefruit EO (0.1%), and rosemary cineole EO (0.05%). Five mixes (one for each oil and one control) of frozen berries weighing 12.5 g were used (three raspberries, four blackberries, four blueberries, four currants were in each mixture). After thawing, the berry blends were inoculated with HAV (ATCC/HM strain) at a concentration of 106.74 TCID50/mL. The inoculated samples were air-dried in a laminar chamber. Then, the samples were placed for 1 h in 20 mL of working solution (66% Tris/glycine/beef extract buffer (TGBE) pH 9.5, 33% peanut oil 0.1% Tween 80) with four EOs at room temperature. A statistically significant reduction in HAV titer on the fruit surface was found after the action of lemon oil (2.84 log TCID50/mL) at a concentration of 0.5%, grapefruit oil (2.89 log TCID50/mL) at a concentration of 0.1%, and rosemary cineole (2.94 log TCID50/mL) at a concentration of 0.05%. The effect of orange essential oil, although it reduced HAV infectivity by >2 log TCID50/mL, was not statistically significant compared to the control. Rosemary cineole worked best, followed by lemon EO, then grapefruit EO. The main active substances in rosemary cineole EO are 1.8 cineole (51.79%), ɑ-pinene (16.54%), camphor (8.38%), and camphene (4.27%), in lemon EO-limonene (71.18%), β-pinene (8.76%), and γ-terpinene (8.24%), in grapefruit EO-limonene (93.45%). Research results indicate that essential oils are more effective in deactivating HAV (by about 2–3 logs) compared to the chlorine disinfection methods described so far.

Fadia S. et al. [92] used essential oils from the flower heads of *Chrysanthemum indicum* and *Chrysanthemum morifolium* in their research. In the flowers of *Chrysanthemum indicum*, 64 active substances were detected, mainly camphor (36.69%), isoborneol (7.64%), α-terpinene (5.73%), and caryophyllene oxide (5.46%). Fifty-five active substances similar to those in *C. indicum* were detected in *Chrysanthemum morifolium*. The dominant constituents were camphor (14.56%), curcumene (10.50%), τ-eudesmol (8.92%), pentacosane (8.65%), borneol (7.95%), and copaene (5.61%). The research was carried out directly in the cell cultures. EO concentrations ranged from 1 to 1000 µg/mL. A plaque reduction test was used to determine the antiviral activity of the essential oils. Vero cells (CCL-81) were grown for 24 h in 5% CO_2_ at 37 °C and then inoculated for 1 h with viruses: herpes simplex type 1 HSV-1 (ATCC VR-1493), hepatitis A HAV virus (ATCC VR-1357), VSV vesicular stomatitis virus (ATCC VR-1238). Infected cells (2 × 103 PFU) were washed and incubated with several different concentrations of EO and aciclovir (positive control) for 1 h. The IC50 value for the oil derived from *C. indicum* was 3.38 µg/mL, while the IC50 for aciclovir was 1.84 µg/mL. Both EO and *C. indicum*, in particular, showed concentration-dependent antiviral activity. They can be used as spices in foods and can be added to a variety of food products and pharmaceutical preparations as natural preservatives with antioxidant potential as both oils showed antioxidant potential with IC50 values of 2.21 and 2.59 mg/mL, respectively, for *C. indicum* and *C. morifolium*.

Sanchez et al. [93] conducted studies in which the effects of three essential oils in various concentrations were assessed: oregano EO (0.5, 1, 2%), thymus EO (0.1; 0.5; 1; 2%), and zataria EO (0.01; 0.05; 0.1%). The HAV strain HM-175 (ATCC VR-1402) was used. The oils were added at various concentrations to the virus suspensions (titer approx. 6 log TCID50/mL) in DMEM with 2% FCS and then incubated at 37 °C in a water bath, shaking at 150 rpm for 2 h. Ten-fold dilutions of the essential oil treated and untreated virus suspensions were inoculated onto monolayers on 96-well plates. Treatment of EO with oregano and zataria resulted in a slight decrease in HAV titer with a maximum reduction of less than 0.5 log TCID50/mL at 0.1% EO Zataria. Thymol at concentrations of 1 and 2% reduced to 1.66 and 2.45 log TCID50/mL, respectively. For thymol on HAV, no effect was observed at any of the concentrations tested.

The role of purified natural products in the prevention and treatment of countless diseases of bacterial, fungal, and viral origin cannot be overestimated. New antiviral drugs have been obtained from natural sources and transformed into preparations for prophylactic and therapeutic purposes [91]. Flavonoids, polyphenols, saponins, proanthocyanins, polysaccharides, organic acids, proteins, polypeptides, and essential oils derived from plants, animals, or microorganisms can control and combat foodborne viral infections, including hepatitis A [94]. Over the past two decades, much effort has been made to identify natural products, mostly of plant origin, to combat foodborne viruses. Natural plant extracts have several potential uses, not limited to increasing the safety of food products and improving their quality, but also as natural antiviral agents [95].

Green tea extract (GTE) is made from the leaves of the evergreen green tea *Camellia sinensis* L. of the *Theaceae* family [96]. Studies have shown that GTE has inhibitory properties against a wide variety of foodborne pathogens [97,98]. Important chemical components of GTE are catechins belonging to the flavonoids [99], which have antibacterial properties against a wide spectrum of Gram-positive and Gram-negative bacteria [98]. In the studies of Randazzo et al. [96], the HAV virus (HM-175/18f strain) was multiplied on FRhK-4 cells cell lines. Various concentrations of green tea extract (from 0.5–10 mg/mL) were added to the cell line wells and incubated for 2 h in 5% CO_2_. DMEM (Dulbecco’s modified Eagle’s medium) was then added to the cells and incubated for 2–15 days. The cytotoxic effect was determined by observation under an optical microscope. One of the objectives of the study was to determine the effectiveness of GTE on the HAV suspension at a concentration of 5 log TCID50/mL for 2 h at 37 °C in PBS with different pH values (5.5, 6.5, 7.2, 8.0, and 8.5). Another aim of the research was to determine the suitability of GTE for disinfecting stainless steel and glass surfaces. The antiviral quality of GTE proved effective in the surface disinfection tests as a 1.5 log reduction and complete deactivation for HAV were recorded on stainless steel and glass surfaces treated with 10 mg/mL GTE for 30 min, analyzed according to ISO 13697: 2001. The next aim was to determine the suitability of GTE as a virucidal agent for washing vegetables. Pieces of fresh lettuce and spinach were inoculated with HAV, and then treated with GTE extract at a concentration of 5 and 10 mg/mL for 15 and 30 min. The HAV titers in lettuce and spinach were significantly reduced after 30 min of treatment with GTE at a concentration of 10 mg/mL. Studies have shown that GTE reduces the number of HAV cells depending on pH. GTE at a concentration of 5 mg/mL reduced the HAV titer by more than 1 log TCID50/mL after 2 h exposure at 37 °C in slightly acidic solutions (pH 6.5). The same concentration of GTE resulted in complete deactivation of HAV and MNV (below the detection limit of 1.15 log TCID50/mL) at neutral (7.2) and slightly basic (8 and 8.5) pH. GTE at a concentration of 0.5 mg/mL showed generally poor inhibition in the tested pH range, with a slight reduction in HAV titer at alkaline pH (8 and 8.5). Research indicates that GTE can be used to control foodborne viral infections by using it to disinfect food products prior to consumption.

In turn, the studies by Falcóa et al. [100] used the HAV HM-175/18f strain (ATCC VR-1402) and FRhK-4 cells. GTE (Naturex SA, Avignon, France) was dissolved in PBS (pH 7.2) and incubated for 24 h (aged GTE) to increase antiviral activity. The GTE was then mixed with an equal volume of MNV-1 and HAV suspensions (approximately 6 or 5 log TCID50/mL, respectively) and incubated at 25,40,50 or 63 °C for 30 min. The antiviral activity was determined by comparing the number of virus particles in suspension with and without GTE. In the next study, fruit juices J1 (with the addition of strawberries, carrots, beetroot, and apples—pH 4.1) and J2 (from apples—pH 3.75) were used. The juices were artificially inoculated with MNV-1 (about 6 log TCID50/mL) and HAV (about 6 log TCID50/mL) and mixed in the same volume with GTE (aged GTE) (10 mg/mL) and incubated for 30 min at 25, 40, 50 and 63 °C. The controls included only juices (cytotoxicity controls) and juices inoculated with viruses without the addition of GTE. There were no significant differences (*p* > 0.05) compared to the control in HAV suspensions subjected to heat treatment, even after heating at 63 °C for 30 min. HAV titers were not significantly lowered (*p* > 0.05) by any of the factors (GTE, temperature) used alone or in combination. The action of GTE and temperature were important in reducing MNV.

*Hibiscus sabdariffa*, belonging to the Malvaceae family, is an annual tropical or subtropical shrub species found in countries such as Mexico, Sudan, India, and Thailand. The calyx of flowers of this plant was found to be rich in bioactive compounds, such as anthocyanins, saponins, phenolic acids, organic acids, and alkaloids [101]. Active substances contained in H. sabdariffa calyx are believed to have a wide range of health properties, including antioxidant, anticancer, cardioprotective, antidiabetic, and antibacterial [102,103]. Protocatechic acid (PCA), the basic component of *H. sabdariffa*, has been shown to be the component responsible for its antibacterial quality [104]. Another chemical component of Hibiscus plants, known as ferulic acid (FA), has also been reported to exhibit antimicrobial properties. The aim of the research by Snehal et al. [105] was to determine the antiviral effect of aqueous extracts of *H. sabdariffa* (HE) and hibiscus components such as PCA (protocatechuic acid), and FA (ferulic acid). Hibiscus flowers were chosen and the HAV-HM175 strain was grown on the fetal rhesus monkey kidney cell line (FRhK4) for 8 days in 5% CO2. The virus (5 log PFU/mL) was incubated with 40 or 100 mg/mL of aqueous hibiscus extract (HE; pH 3.6), protocatechic acid (PCA; 3 or 6 mg/mL, pH 3.6), ferulic acid (FA; 0.5 or 1 mg/mL; pH 4.0), malic acid (10 mM; pH 3.0), and PBS (pH 7.2 as control) at 37 °C for 24 h. All studies were performed in three replications, and plaque reduction in two replications. The HAV titer was reduced to an undetectable level by both HE concentrations after 24 h. HE at 40 mg/mL reduced the HAV titer by 1.29 ± 0.05 and 1.14 ± 0.01 log PFU/mL after 3 and 6 h, respectively, and to an undetectable level only after 24 h. HE at 100 mg/mL reduced the HAV titer by 1.37 ± 0.02 and 1.33 ± 0.01 log PFU/mL after 3 and 6 h, respectively, and to an undetectable level only after 24 h. PCA at a concentration of 1.5 mg/mL lowered the HAV titer by 0.26 ± 0.01 and 0.25 ± 0.01 log PFU/mL after 6 and 24 h, respectively, and at a concentration of 3 mg/mL by 0.42 ± 0.06 and 0.74 ± 0.06 log PFU/mL after 6 and 24 h, respectively. FA concentrations of 0.5 and 1 mg/mL did not reduce the HAV titer even after 24 h. Malic acid also did not reduce the HAV titer, even after 24 h. Transmission electron microscopy did not show conclusive results. The results suggest that *H. sabdariffa* extracts have the potential to prevent the spread of foodborne viruses. The consumption of hibiscus tea is becoming a popular trend all over the world, and *H. sabdariffa* extracts are widely used in the preparation of teas with medicinal properties.

The results of research by Snehal et al. [105] show promise for the use of aqueous *H. sabdariffa* extracts as food and drink additives or direct consumption products to reduce or alleviate the symptoms of foodborne viral diseases.

On the other hand, in the studies conducted by El-Shiekh et al. [106], the alcoholic extract of *Hibiscus schizopetalus* showed only weak activity against HAV.

Blueberries and berry extracts are known for their health and antimicrobial properties. Natural therapeutic or prophylactic approaches to reduce the incidence of foodborne viral diseases are currently of interest to many authors, and the antiviral activity of blueberries and berry extracts was assessed in the studies of Snehal et al. [107] using standard plaque tests at 37 °C for 24 h. Viruses with a titer of ~5 log PFU/mL were mixed with equal volumes of blueberry juice (BJ) (pH 2.8), neutralized BJ (pH 7.0), blueberry proanthocyanidins (BB-PAC) (1, 2, 4, and 10 mg/mL), malic acid (pH 3.0) or PBS (pH 7.2) and incubated for 24 h at 37 °C. Each experiment was performed in duplicate. HAV titers dropped to undetectable levels after 30 min with 2 and 5 mg/mL BB-PAC, after 3 h with 1 mg/mL BB-PAC and by ~2 log PFU/mL at BJ after 24 h.

Grape seed extract (GSE), vitis vinifera, is obtained as a by-product of the production of juice and wine during the processing of grapes [108]. Currently, it is known that it has a variety of bioactive compounds, including anthocyanins, flavonoids, proanthocyanidins, polyphenols, procyanidins; and resveratrol, a derivative of stylbene [109]. The antioxidant, anti-inflammatory, cardioprotective, hepatoprotective, neuroprotective, and antimicrobial properties of these compounds have resulted in the extract having pharmacological and therapeutic properties (Xia 2010). The antiviral activity of GSE against some foodborne viruses, including hepatitis A virus (HAV), has been described in the literature.

The studies by Snehal et al. [110] used the HAV-HM175 strain and the FRhK4 cell line. The research was carried out in model food systems as well as in artificial conditions similar to those in the stomach. The prepared GSE suspensions at concentrations of 2, 4, and 8 mg/mL were mixed with the appropriate volume of the HAV virus suspension to obtain a virus titer of ~5 log PFU/mL. Then, the HAV suspension was mixed with equal volumes of GSE (2, 4, and 8 mg/mL) with apple juice (AJ; pH 3.6 or pH 7.0) or 2% milk and then incubated at 37 °C for 5, 15, 30, 60, 120, 180, and 360 min. Virus survival was assessed using standard plaque tests. TEM was used to determine any structural and/or morphological changes in GSE treated and untreated viruses. GSE at a concentration of 1, 2, and 4 mg/mL in AJ (pH 3.6) reduced HAV to an undetectable level after 1 h at 37 °C, while AJ alone without GSE caused a reduction of 0.63 ± 0.20 log PFU/mL after 24 h. Concentrations of 1, 2, and 4 mg/mL GSE in milk did not cause a significant difference in the HAV titer after 24 h. For artificial intestinal fluid studies, the HAV particle number was reduced to undetectable levels after exposure to GSE at 1 mg/mL after 6 h at 37 °C.

A 2011 study showed that GSE decreased HAV titer in a dose-dependent manner, wherein increasing GSE concentrations resulted in a greater reduction in viral titers [111]. It should be noted that the effectiveness of GSE was also influenced by the temperature and the level of the virus. At 37 °C, the reduction corresponded to the concentration, 0.25 mg/mL GSE, 0.5 mg/mL GSE, 1 mg/mL GSE with a high HAV titer (about 7-log10-PFU): 1.81; 2.66 and 3.20 and low (ca 5-log10-PFU): 1.86; 2.26 and 2.89. However, significant differences were visible at room temperature, wherein the reduction was, respectively, to the concentration, 0.25 mg/mL GSE, 0.5 mg/mL GSE, 1 mg/mL GSE with a high HAV titer (about 7-log10-PFU): 0, 86; 1.22 and 1.90 and low (approximately 5-log10-PFU): 2.40; 2.62 and 3.01. The greatest effectiveness of GSE was demonstrated at 37 °C in a concentration of 1 mg/mL, with a high titer HAV, and the lowest at a concentration of 0.25 mg/mL at room temperature, also with a high HAV titer. Further studies by these researchers showed that GSE at concentrations ranging from 0.25 mg/mL to 1 mg/mL reduced viral contamination levels in food products without causing significant changes in the color of lettuce and peppers (Su 2013). However, it was found that the reduction caused by GSE was dependent on the virus type, and the HAV titer was reduced by GSE at concentrations ranging from 0.25 to 1.0 mg/mL by about 1 log10 PFU during 1 min of exposure.

Cinnamaldehyde (CNMA) is an organic compound that gives cinnamon its flavor and aroma. The studies by Fabra et al. [112] used the HAV-HM-175/18f strain, which was grown on FRhK-4 cells. Various concentrations of cinnamaldehyde were used—0.1, 0.5, and 1%—the virus titer was initially 6–7 log10 TCID50/mL, and incubation was carried out for 2 h at 37 °C and 5% CO_2_. The cytotoxic effects were determined both visually under an optical microscope and with the Vybrant^®^ MTT cell proliferation assay (Thermo Fisher Scientific, Waltham, MA, USA) according to the manufacturer’s instructions. Each CNMA suspension was diluted with 50% ethanol and the same concentration of virus particles was added to them. Each experiment was performed in triplicate. The antiviral activity of CNMA was assessed by comparing the titer of viruses in the suspensions without CNMA and the viral suspensions treated with CNMA, which was found to be effective in reducing the titers of norovirus and HAV surrogate viruses as a function of duration and temperature. Incubation in CNMA at 4 °C for 2 h had no effect on the virus titer. Incubation with 1% CNMA reduced HAV titers by 1 log10 TCID50/mL. Incubation overnight at 37 °C with CNMA was effective in reducing HAV viral loads in a dose-dependent manner, wherein increased CNMA concentrations resulted in increased reductions in viral titers. The initial viral load was 6.03 ± 0.94, after an overnight exposure to CNMA at a concentration of 0.1, 0.5 and 1 mg/mL at 37 °C, it decreased to 6.00 ± 0.19, 3.32 ± 0.12, 2.66 ± 0.07.

Ginseng (Panax ginseng Meyer) contains numerous bioactive ingredients, including ginsenosides, phytosterols, polysaccharides, polyacetylenes, polyacetylene alcohols, fatty acids, and peptides. It has anti-stress, anti-carcinogenic, anti-inflammatory, antioxidant, antibacterial, antiviral, and antifungal properties [113]. During processing, red ginseng is usually steamed and fermented with the ginseng rind, which affects the composition of the saponin contained. Due to its various bioactive functions, red ginseng is widely used as an oriental medicinal herb and food ingredient [114]. The aim of the study was to investigate the antiviral effect of red ginseng extract and ginsenosides on the hepatitis A virus (HAV). To test the antiviral activity against HAV, FRhK-4 cells (the fetal rhesus monkey kidney) infected with HAV were treated with red ginseng and purified ginsenosides Rb1 and Rg1. The HAV titer dropped significantly in all groups which had previously received either red ginseng or purified ginsenosides. The results showed that red ginseng and ginsenosides Rg1 and Rb1 can lower HAV titers. The authors suggest that regular consumption of red ginseng as a dietary supplement may help prevent HAV infections.

The Egyptian Red Sea seagrass, *Thalassodendron ciliatum*, is characterized by high levels of “tanning cells” and a high phenolic content. Compounds isolated from raw seagrass extract have been shown to have antioxidant and cytotoxic effects [115,116].

Hamdy et al. [116] studied the antiviral quality of extracts from seagrass samples of *Thalassodendron ciliatum*. Fresh *T. ciliatum* (800 g) was blended in an electric blender with methanol, and the process was repeated until exhaustion. The combined extracts were filtered, and the solvent was evaporated under reduced pressure at 45 °C. The crude extract was partitioned between ethyl acetate (EtOAc) and H_2_O several times. The ethyl acetate fraction (10.61 g) was chromatographed on a Sephadex LH-20 column (600 mm) with step gradient elution starting from 30% ethanol in H_2_O to 100% ethanol. Fractions of 250 mL each were collected and those exhibiting similar TLC profiles were combined. Subfractions were subsequently fractionated on Sephadex LH-20 columns with different elution systems to allow the purification and identification of six phenolic compounds: rutin, asebotin, 3-hydroxyasebotin, quercetin-3-O-β-D-xylopyranoside, a racemic mixture of (+)-catechin and (–)-catechin, and trans-caffeic acid. The total extract, as well as the isolated pure compounds, were tested against HAV. The antiviral quality of the samples was determined at non-cytotoxic concentration by the plaque assay method. The crude extract showed 100% inhibition of hepatitis A (HAV) at the lowest concentration tested (20 μg/mL). The antiviral activity of the crude extract against HAV was lost by fractionation, which could be explained by the synergistic action of several compounds in the crude extract [116].

The antiviral effect of licorice root has been described. The main chemical components of licorice root are triterpene saponins. Glycyrrhizin is the main ingredient, with concentrations ranging from 1% to 9%, depending on the species, geography, and extraction method. Glycyrrhizin is a glycoside which occurs as a mixture of the calcium, sodium, and potassium salts of glycyrrhizinic acid (also called licorice acid). During hydrolysis, two molecules are released: D-glucuronic acid and Aglycone of 18-β-glycyrrhetinic acid (also called licorice acid), a pentacyclic triterpenoid of the β-amyrin type [117]. However, there are no recent reports on the efficacy of licorice against HAV. Table 1 shows the composition and source of the essential oils and plant substances presented in the literature used in this review.

## 5. Conclusions

Viruses, including HAV, may cause foodborne diseases. As there is an increasing demand for food that does not contain synthetic chemicals as preservatives, it is imperative to research and identify alternative and safe methods for protecting food products. Even though many natural products are produced today to preserve and extend the shelf life of foodstuffs, there are still many unexplored sources. The natural compounds from the by-products of plants, algae, and fungi are now seen as possible compounds to use as new antimicrobial agents. More research is needed to determine the optimal concentrations of antimicrobials that can be safely used in food without unduly altering any sensory characteristics. The antiviral effect of essential oils, including HAV, as well as extracts and plant substances depends on the concentration of the active substance, its duration of action, and temperature.

Essential oils and extracts are safe and environmentally friendly, and many essential oils have anti-inflammatory, antibacterial, antifungal, antiviral, and antiseptic properties. EO plays an important role in food processing due to the myriad of characteristics mentioned above.

## Figures and Tables

**Table 1:** Main compounds of essential oils and plant substances.

Name of Plant Source	Substance/ Essential Oil (EO)/Plant Extract	Reduction in HAV Titer	References
Citrus limon (lemon), Citrus sinensis (sweet orange), Citrus paradisi (grapefruit), Rosmarinus officinalis (rosemary cineole)	Lemon EO: limonene (71.18%), β-pinene (8.76%), and γ-terpinene (8.24%) Sweet orange EO: limonene 95.74%) Grapefruit EO (limonene 93.45%) Rosemary cineole EO: 1.8 cineole (51.79%), ɑ-pinene (16.54%), camphor (8.38%), and camphene (4.27%))	2.84 log TCID50/mL >2 log TCID50/mL 2.89 log TCID50/mL 2.94 log TCID50/mL	[59]
Chrysanthemum indicum, Chrysanthemum morifolium	EO from flower heads, major constituents: camphor, borneol, camphene, α-pinene, p-cymene and 1.8 cineole,	2.21 log PFU/mL ID50 2.59 log PFU/mL ID50	[92]
Origanum vulgare Thymus vulgaris Zataria multiflora	Oregano EO—carvacrol Thyme EO—thymol Zataria EO—carvacrol, thymol methyl ether	<0.5 log TCID50/mL 1.66 (1%); 2.45 (2%) log TCID50/mL <0.5 log TCID50/ml	[93]
Green tea	Green tea natural extract—soluble in water—content of epigallocatechin-3-gallate: 40–50%	1 (pH 6.5), 1.15 (pH 7.2) log TCID50/mL	[96,100]
Hibiscus sabdariffa	Hibiscus sabdariffa extract: PCA (protocatechuic acid), FA (ferulic acid), MA (malic acid)	1.29 ± 0.05–1.14 ± 0.01 (40mg/mL HE) log PFU/mL 1.37 ± 0.02–1.33 ± 0.01 (100mg/mL HE) log PFU/mL	[105]
Blueberry	Blueberry and blueberry extracts, blueberry juice (BJ) and blueberry proanthocyanidins (BB-PAC, B-type PAC structurally different from A-type PAC found in cranberries)	1–2 log PFU/mL	[107]
Grape seed	Grape seed extract (GSE), Gravinol S, proanthocyanidins	0.63 ± 0.2 log PFU/mL 1.86 log PFU/mL (0.25 mg/mL GSE), 2.26 log PFU/mL (0.5 mg/mL GSE); 2.89 log PFU/mL (1 mg/mL GSE)	[110,111,113]
Cinnamon	Cinnaaldehyde (CNMA)—3-Phenylprop-2-enal; ≥95% purity	1 log10 TCID50/mL	[112]
Hibiscus schizopetalus	Dichloromethane Fraction (DCM-F), n-Butanol Fraction (Bu-F)	1–2 log PFU/mL	[106]
Panax ginseng Meyer	Ginsenosides, saponin	0.23 ± 0.48 to 0.57 ± 0.25 log 10 PFU/mL 0.45 ± 0.46 to 0.66 ± 0.52 log 10 PFU/mL	[114]
Thalassodendron ciliatum	Flavonoids: rutin, asebotin, 3-hydroxyasebotin, quercetin-3-O-β-D-xylopyranoside, and a racemic mixture of catechin	100% (crude extract)	[116]
Glycyrrhiza	Glycyrrhizin	No data	[117]

## Data Availability

Not applicable.
